# The Natural Variation of Seed Weight Is Mainly Controlled by Maternal Genotype in Rapeseed (*Brassica napus* L.)

**DOI:** 10.1371/journal.pone.0125360

**Published:** 2015-04-27

**Authors:** Na Li, Wei Peng, Jiaqin Shi, Xinfa Wang, Guihua Liu, Hanzhong Wang

**Affiliations:** 1 Oil Crops Research Institute of the Chinese Academy of Agricultural Sciences, Key Laboratory of Biology and Genetic Improvement of Oil Crops, Ministry of Agriculture, Wuhan, Hubei Province, 430062, China; 2 College of Life Science, Hubei University, Wuhan, Hubei Province, 430062, China; Wuhan University, CHINA

## Abstract

Seed weight is a very important and complex trait in rapeseed (*Brassica napus* L.). The seed weight of rapeseed shows great variation in its natural germplasm resources; however, the morphological, cytological and genetic causes of this variation have remained unclear. In the present study, nine highly pure inbred rapeseed lines with large seed weight variation and different genetic backgrounds were selected for morphological, cytological and genetic studies on seed weight. The results showed the following: (1) Seed weight showed an extremely significant correlation and coordinated variation with seed size (including seed diameter, seed surface area and seed volume), but it showed no significant correlation with bulk density, which suggests that seed weight is determined by size rather than bulk density. (2) Seed weight showed a higher correlation with the cell numbers of seed coats and cotyledons than the cell sizes of seed coats and cotyledons, which suggests that cell number is more tightly correlated with final seed weight. (3) Seed weight was mainly controlled by the maternal genotype, with little or no xenia and cytoplasmic effects. This is the first report on the morphological and cytological causes of seed weight natural variation in rapeseed. We concluded that the natural variation of seed weight is mainly controlled by maternal genotype. This finding lays a foundation for genetic and breeding studies of seed weight in rapeseed and opens a new field of research on the regulation of seed traits in plants.

## Introduction

Seed weight is an important trait with respect to plant evolution and crop improvement. Small seeds are easily dispersed, whereas large seeds usually have better adaptability under biotic and abiotic stresses and produce seedlings that may have superior competitive survival rates [1]. However, limited resources in the mother plant generally lead to a tradeoff between the seed number and weight [2]. In agriculture, seed weight is one of the main components affecting seed yield [3] and is a target of artificial selection [4].

The seed weight of most crops has different degree of natural variation, which usually harbores favorable alleles that are invaluable for crop improvement. Since the beginning of agriculture, seed weight/size has been subjected to artificial selection, and therefore, most crop plants have seeds larger than those of their wild relatives [5]. Investigations on the natural variation of seed weight will provide insights into its improvement.

From a genetic perspective, seed weight is a very complex trait. The seed is the result of double fertilization; thus, it consists of three components: a diploid embryo, triploid endosperm and diploid maternal ovule [6]. In addition, the development of a seed is dependent on the nutrients supplied by the mother plant [7]. Therefore, the genetic model of the seed in theory includes the effects of the maternal genotype and those of the cytoplasm, embryo and endosperm [8], which can be attributed to maternal and xenia effects. The relative contributions of maternal and xenia effects are of fundamental interest in genetic and breeding studies [9]. However, these effects have not yet been investigated for seed weight in rapeseed (*Brassica napus* L.).

From a morphological perspective, the final seed weight is determined by the bulk density and seed volume, which are determined by the cell size and cell number. The weight of seeds shows a large variation (≈4-fold) in rapeseed natural germplasm resources [10, 11]. However, whether this variation is caused by changes in seed volume and/or bulk density as well as changes in cell size and/or cell number remains unexplored.

In this present study, the natural variation of seed weight in rapeseed was systematically investigated at the morphological, cytological and genetic levels. The main objectives were to (1) select several representative rapeseed inbred lines for seed weight study, (2) investigate the morphological and cytological causes of the natural variation in seed weight, (3) determine the maternal and xenia effects on the natural variation of seed weight using a subtle experimental design, and (4) estimate the effects of maternal genotype and cytoplasm on seed weight.

## Materials and Methods

### Plant material

A collection of 576 [11] and 487 (S1 Table) rapeseed inbred lines/cultivars was developed by our lab. This collection was genotyped using 101 published SSR (simple sequence repeat) markers (S2 Table) [12–16] that are evenly distributed across all 19 rapeseed chromosomes and phenotyped for tens of traits. From these lines, four large-seed (No. 02454, No. 09131, No. 19004 and Qing662) and five small-seed (No. 01201, No. 02210, No. 03482, No. 19179 and No. 91032) rapeseed inbred lines with broad genetic diversity and similar flowering times were selected.

### Genetic diversity analysis

DNA extraction and genotyping were performed as described by Li et al. [11]. The gene diversity, observed heterozygosity, polymorphic information content (PIC) and Nei’s genetic distance [17] among the lines were calculated using Powermarker version 3.25 [18]. A dendrogram was constructed based on the UPGMA algorithm (unweighted pair group method with arithmetic average) implemented in NTSYSpc 2.1 [19]. The relative kinship coefficients were calculated using the SPAGedi software package [20]. All negative values between lines were set to 0 [21].

### Microscopic analysis

For the determination of cell number and cell size in the outer layer of the seed coat and the cotyledon, mature seeds were soaked in distilled water for 24 h and dissected to isolate the seed coat and embryo. Then, the seed coat and embryo were fixed overnight with FAA solution [22], which contained 5% (v/v) acetic acid, 45% (v/v) ethanol, and 5% (v/v) formaldehyde. The seed coats were rendered transparent by incubation overnight (12–24 h) in a chloral hydrate solution as described previously [23]. After dehydration in an ethanol series (50%, 70%, 95% and 100%), the embryos were infiltrated and subsequently embedded in paraffin wax according to the methods of a previous study [24]. Sections were obtained using a Leica RM 2016 microtome (Leica, Nanterre Cedex, France) and stained with safranin fast green. Observations were performed using a light fluorescence microscope (Olympus IX-71, Tokyo, Japan). Cleared cells were photographed, and the mean cell area of at least 10 cells was determined based on four individuals for each line using the Image J program (http://rsb.info.nih.gov/ij/). The numbers of cells in the region of the outer seed coat and the outer epidermis cotyledon were determined.

### Maternal effect study

For the maternal effect study, we performed the first experiments using improved diallel crossing with minimal environmental influences as described by Wang et al. [25] using two large-seed lines (No. 02454 and No. 09131) and three small-seed lines (No. 02210, No. 03482 and No. 91032) in Aug. 2012 in Xining (code X12). In Mar. 2013, we performed the second experiments after adding two large-seed lines (No. 19004 and Qing662) and two small-seed lines (No. 01201 and No. 19179) in Wuhan (code W13). The improved aspects were as follows: First, both of the F_1_ hybrid seeds and self-pollinated seeds were produced by emasculation and artificial pollination. Second, selfing and crossing were performed alternately on different branches of the same mother plant. The details were as follows: For the large-seed line (L) × small-seed line (S) cross, two plants (A and B) from the L were selected as the maternal pollen acceptor and the branches of each plant were numbered from the first to the fourth. When pollinating, the first and third branches of plant A were self-pollinated (L × L), whereas the second and fourth branches were cross-pollinated by the small-seed line (L × S). At the same time, the first and third branches of plant B were cross-pollinated by the small-seed lines (L × S), whereas the second and forth branches were self-pollinated (L × L). Reciprocal crosses for the small-seed line (S) × large-seed line (L) were performed in the same way. Each cross was repeated at least three times, and pollinations were completed within one day.

### Field experiment and measurement of traits

Each line was sown in ten rows with nine plants per row with a spacing of 40 × 16.7 cm. The F_1_ seeds of L × S and S × L crosses were arranged in a randomized complete block design with three replications (codes W13 and W14). Each block contained one row with 15 plants with spacing of 33.3 × 16.7 cm. The seeds were sown by hand, and the field management followed local standard agricultural practice.

For the nine rapeseed inbred lines, seed weight was measured based on 1000 fully developed seeds from the main raceme (open pollination) of at least 15 representative individuals. Seed diameter (d) was measured using vernier calipers based on at least 30 seeds for each individual. With each rape seed considered to be an approximate sphere [10, 26], the seed surface area, seed volume and bulk density were estimated using the following equations: seed surface area = 4π(d/2)^2, seed volume = (4/3) π(d/2)^3, bulk density = seed weight/(seed volume * 1000), where d is the seed diameter. For F_1_ seeds (artificial selfing and crossing) from mother plants, branches from healthy plants were harvested and threshed individually. Seeds from different branches of the same treatment were averaged to determine the seed weights of the F_1_ selfed and crossed seeds. For the F_2_ seeds from the reciprocal crosses, the seeds from the main raceme (open pollination) of at least 15 individuals were averaged to determine the seed weight.

### Statistical analysis

Pearson’s correlation coefficients were calculated using the SAS CORR procedure. Significant differences among the weights of the F_1_ selfed and crossed seeds and the reciprocal F_2_ seeds were estimated using one way ANOVA analysis in the SAS software [27]. The maternal effect on seed weight was estimated based on a previously described method [25, 28]. The seed weights of the F_1_ hybrid seeds were calculated as follows: F_1_ = mP_1_ + (1-m) P_2_, where, F_1_ is the seed weight of the F_1_ hybrid seeds, m (the maternal effect value) = ∑(F_1_-P_2_)(P_1_-P_2_)/ ∑(P_1_-P_2_)^2^, and P_1_ and P_2_ are the seed weights of the maternal and male parents (artificially selfed), respectively.

An embryo-cytoplasm-maternal (GoCGm) model for diploid seeds was employed to determine the main genetic effects and their *GE* interaction effects [8, 29] using QGAStation 1.0 (http://ibi.zju.edu.cn/software/qga/index.htm). Genetic variance components were estimated according to the MINQUE (0/1) method [8]. The main genetic effects and *GE* interaction effects were predicted by the AUP (adjusted unbiased prediction) method. The standard error of estimated variance was analyzed by the jackknife procedure, and the t-test was used to test the significance of statistical parameters.

## Results

### Genetic diversity and relative kinship analysis of the research materials

**Fig 1:**
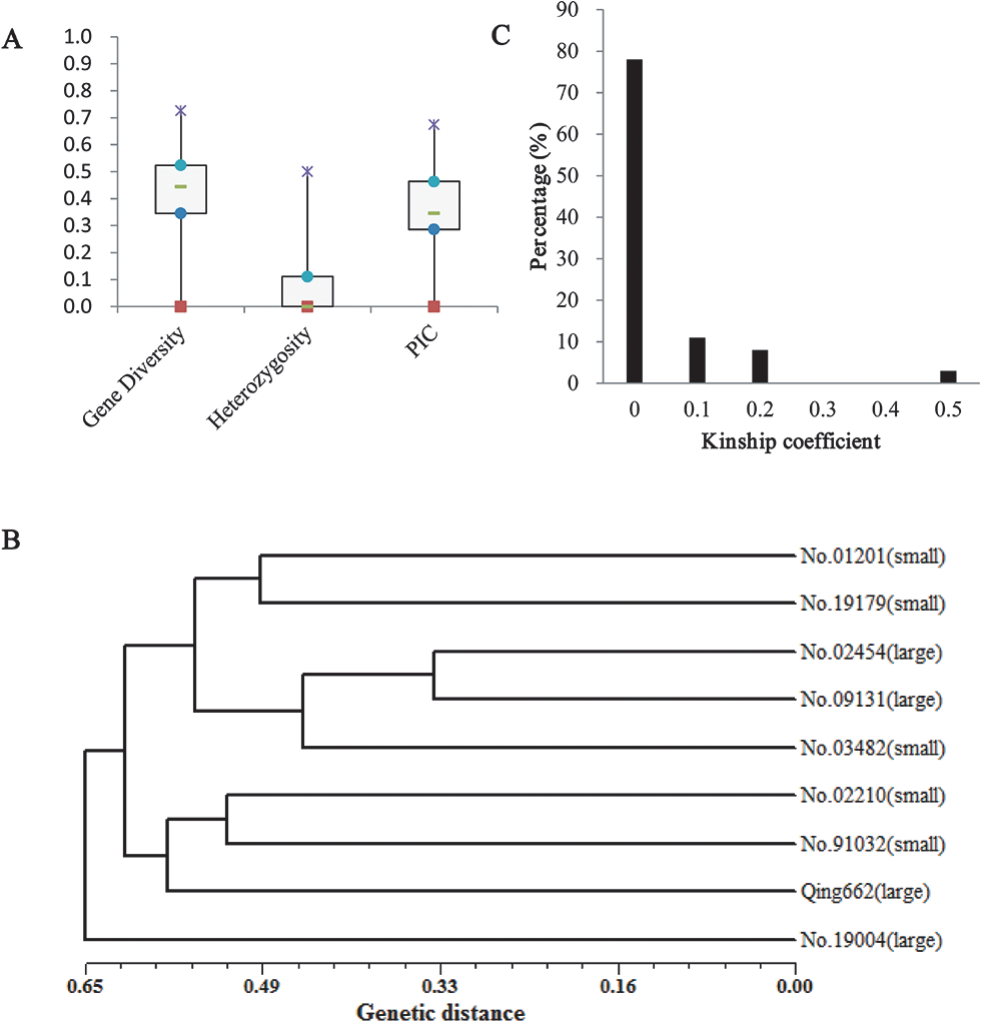
Genetic diversity and relative kinship analysis. A: Box and whiskers summary of statistics for 101 SSR markers in nine rapeseed lines. B: A dendrogram of the nine inbred lines clustered on the basis of Nei's (1972) genetic distance. C: The distribution of kinship coefficients.

To estimate the genetic diversity of the nine inbred lines (Fig 1A), 101 SSR markers were used (S2 Table). The average heterozygosity of the 101 SSR markers was 0.06, and approximately 58% of the SSRs were 0. For all of the studied SSR markers, the gene diversity varied from 0 to 0.73 with an average of 0.41, and PIC varied from 0 to 0.68 with an average of 0.35. Nei’s genetic distances ranged from 0.33 to 0.78 with an average of 0.59 (S3 Table). The two inbred lines with the closest genetic distance were No. 02454 and No. 09131, which had a common ancestor (No. 73290). Nei’s genetic distances matrices were used to build a dendrogram with the UPGMA algorithm (Fig 1B). As expected, No. 02454 and No. 09131 clustered on the same branch. However, there was no relationship between the classification and seed weight, which strongly suggests that the nine rapeseed lines have broad genetic backgrounds. The kinship coefficients ranged from 0 and 0.44 with an average of 0.03 (Fig 1C and S4 Table). As expected, the two inbred lines with the largest kinship coefficient (0.44) were No. 02454 and No. 09131. The kinship coefficients for the remaining pairs of lines were less than 0.2, which demonstrates that most of the nine rapeseed lines were not related or only weakly related.

### Morphological and cytological causes for seed weight variation

**Fig 2:**
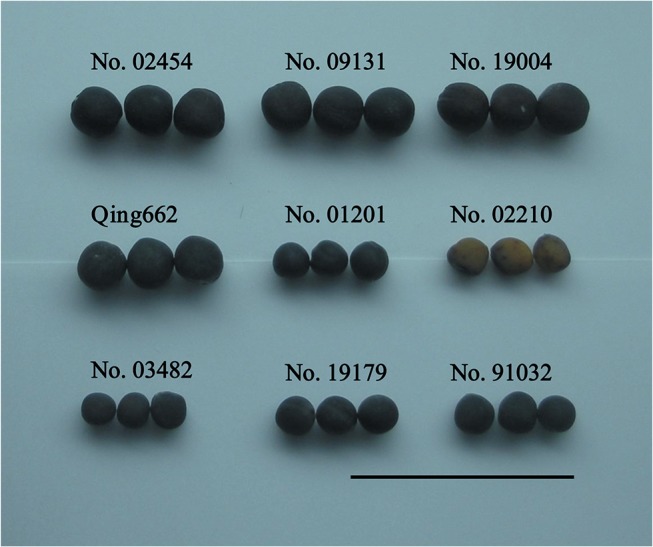
Seeds from nine rapeseed lines. Bar = 1 cm.

To investigate the morphological causes of seed weight variation, we measured the seed diameter and further calculated the seed surface area, seed volume and bulk density for nine rapeseed inbred lines (Table 1). For the large-seed lines, the diameter, surface area, volume, bulk density and weight varied from 2.02 mm to 2.21 mm, 12.87 mm^2^ to 15.36 mm^2^, 4.35 mm^3^ to 5.66 mm^3^, 1.20 mg/mm^3^ to 1.27 mg/mm^3^ and 6.14 g to 7.16 g, with averages of 2.13 mm, 14.28 mm^2^, 5.08 mm^3^, 1.25 mg/mm^3^ and 6.51 g, respectively. For the small-seed lines, the diameter, surface area, volume, bulk density and weight varied from 1.49 mm to 1.65 mm, 7.00 mm^2^ to 8.61 mm^2^, 1.76 mm^3^ to 2.38 mm^3^, 1.16 mg/mm^3^ to 1.32 mg/mm^3^ and 2.34 g to 2.95 g, with averages of 1.58 mm, 7.85 mm^2^, 2.08 mm^3^, 1.24 mg/mm^3^ and 2.63 g, respectively. The diameter, surface area, volume and weight of the four large-seed lines were all significantly greater than those of the five small-seed lines, with average proportions of 34.82%, 81.85%, 144.82% and 147.34%, respectively, whereas bulk density did not differ between the large-seed and small-seed lines (Table 1 and Fig 2). Moreover, the seed diameter, seed surface area and seed volume of these inbred lines showed extremely significant/high correlations with seed weight (p < 0.0001/r = 0.9693, p < 0.0001/r = 0.9743 and p < 0.0001/r = 0.9738, respectively), whereas bulk density showed no correlation with seed weight (r = -0.0336/p = 0.8628).

**Table 1:** Morphological and cytological traits for nine rapeseed inbred lines (mean ± SD).

Cha-racte-ristic	Material code	Seed weight (g)^a^	Seed diameter (mm)	Surface area(mm^2^)	Volume(mm^3^)	Bulk density (mg/mm^3^)	Seed coat cell number	Seed coat cell size (μm^2^)	Cotyledon cell number	Cotyledon cell size (μm^2^)
large seed	No. 02454	6.36±0.37b	2.12±0.02ab	14.17±0.29a	5.02±0.15b	1.27±0.06ab	42917±5767b	336.34±50.33a	19598±1225c	725.61±44.69a
	No. 09131	6.36±0.20b	2.16±0.03a	14.70±0.40a	5.30±0.21ab	1.20±0.03ab	52181±5887a	285.32±35.53c	21760±1971b	680.04±57.13ab
	No. 19004	7.16±0.34a	2.21±0.03a	15.36±0.36a	5.66±0.20a	1.27±0.05ab	49280±3704a	313.35±24.40b	24414±1549a	631.49±40.82b
	Qing662	6.14±0.35b	2.02±0.11b	12.87±1.34b	4.35±0.67c	1.26±0.06ab	44039±2915b	292.90±19.54bc	26063±2655a	497.94±55.44c
	**Mean**	**6.51**	**2.13**	**14.28**	**5.08**	**1.25**	**47104**	**306.98**	**22959**	**633.77**
small seed	No. 01201	2.78±0.22cd	1.65±0.08c	8.61±0.80c	2.38±0.33d	1.17±0.07b	30379±1938d	284.07±17.30c	13404±1359f	647.82±69.10b
	No. 02210	2.55±0.07cd	1.56±0.05cd	7.65±0.44cd	1.99±0.17de	1.29±0.09ab	31434±1629cd	243.67±12.64d	15313±1696e	504.24±52.67c
	No. 03482	2.53±0.09cd	1.57±0.04cd	7.74±0.36cd	2.02±0.14de	1.26±0.10ab	33418±3193cd	233.24±21.36d	15093±1764e	518.34±58.40c
	No. 19179	2.95±0.36c	1.62±0.08c	8.25±0.77cd	2.23±0.31de	1.32±0.07a	34463±3359c	240.87±22.22d	16695±1177de	495.08±33.29c
	No. 91032	2.34±0.13d	1.49±0.15d	7.00±1.33d	1.76±0.48e	1.16±0.00b	30931±2872d	226.79±20.81d	17185±2795d	415.12±70.46d
	**Mean**	**2.63**	**1.58**	**7.85**	**2.08**	**1.24**	**32125**	**245.73**	**15538**	**516.12**

^a^: Columns followed by the same letter are not significantly different at p < 0.05 (Duncan’s test). Data are the mean values of at least three plants from each line.

**Fig 3:**
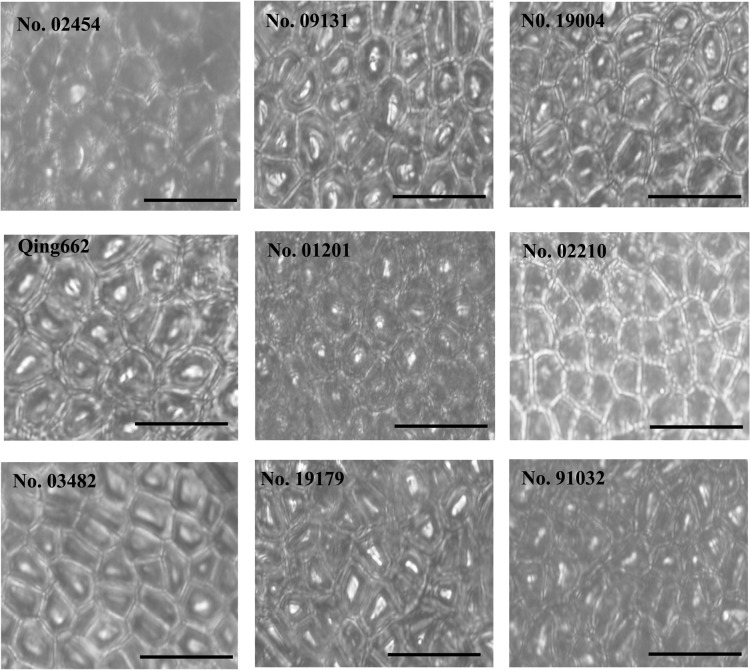
Outer layer cells from the seed coats of the nine rapeseed inbred lines. Bar = 50 μm.

**Fig 4:**
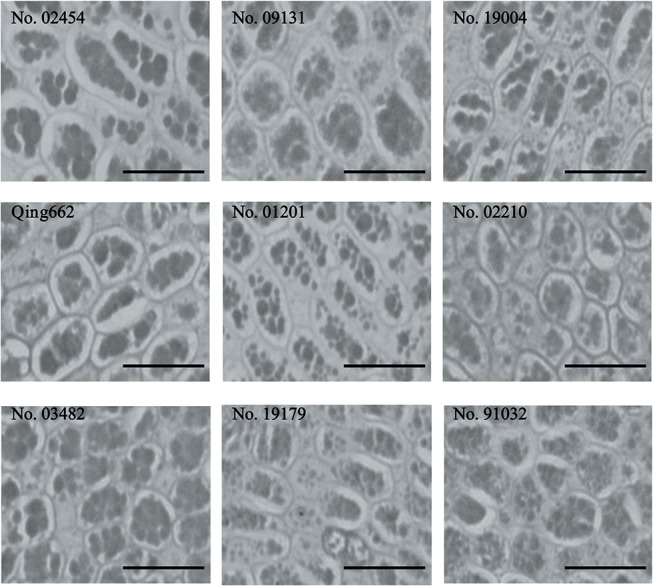
Cells from the embryonic cotyledons of the nine rapeseed inbred lines. Bar = 50 μm.

The cell size and cell number of the large- and small-seed inbred lines were estimated in the outer layer of the seed coat (Fig 3) and the embryonic cotyledons (Fig 4). The seed coat cell sizes, seed coat cell numbers and cotyledon cell numbers of the four large-seed lines were all larger than those of the five small-seed lines, with average proportions of 24.93%, 46.63% and 47.76%, respectively. For cotyledon cell sizes, except for the large-seed line Qing662, which had a smaller cell size, and small-seed line No. 01201, which had a larger cell size, the large-seed lines were significantly larger than those of the small-seed lines by an average of 22.80%. Moreover, the seed coat cell size, seed coat cell number, cotyledon cell size and cotyledon cell number of these lines all showed significant/high correlations with seed weight (p < 0.0001/r = 0.6912, p < 0.0001/r = 0.8600, p < 0.0001/r = 0.5869 and p < 0.0001/r = 0.7894, respectively).

### Maternal and xenia effects on seed weight

**Fig 5:**
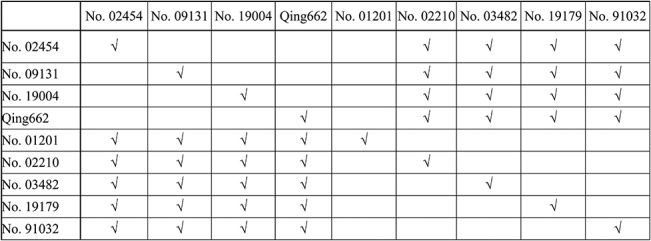
Treatment combinations for the maternal study. “√” represents the combination emasculation hybrid treatment.

A maternal effect study was performed (Fig 5) using the above nine inbred lines with significant differences in seed weight. The mean seed weight of the F_1_ hybrid seeds from crosses with large-seed lines as maternal parents pollinated with small-seed lines was slightly lower (not significantly), by 0.36%, relative to the artificially self-pollinated seeds from large-seed lines. The mean seed weight of the F_1_ hybrid seeds from crosses with small-seed lines as maternal parents pollinated with large-seed lines was higher (not significantly), by 5.30%, relative to the artificially self-pollinated seeds from small-seed lines (Table 2). In other words, the weight of F_1_ hybrid seeds was always similar to that of the maternal parent regardless of whether the maternal parent was from large- or small-seed line. This finding suggests that there is a strong maternal influence on the weight of hybrid seeds. The maternal effect of 20 crosses (two years) involving large-seed lines pollinated by small-seed lines had a mean value of 0.95, and almost half of these were larger than 1 due to the existence of ultra-high seed weight parent individuals. The xenia effect had a mean value of 0.05. Similarly, the maternal effect of 20 crosses involving small-seed lines pollinated by large-seed lines had a mean value of 0.90, whereas the xenia effect had a mean value of 0.10. Overall, these results indicate that the weights of the F_1_ hybrid seeds of rapeseed are mainly controlled by the maternal effect and accompanied by the minor xenia effect.

**Table 2:** Maternal and xenia effects on the weight of hybrid seeds (thousand seed weight).

Parental lines (L-S^a^)	F_1_(L×L)^b^	F_1_(L×S)	Maternal effect^c^	Xenia effect	F_1_(S×S)	F_1_(S×L)	Maternal effect	Xenia effect	Experiment code
No. 02454-No. 02210	6.22±0.45A	6.12±0.70A	0.95	0.05	4.33±0.29B	4.57±0.49B	0.85	0.15	X12
No. 02454- No. 91032	6.51±0.37A	6.37±0.83A	0.99	0.01	4.93±0.72B	5.06±0.19B	0.83	0.17	X12
No. 02454- No. 03482	6.14±0.13A	6.15±0.59A	0.93	0.07	4.99±0.42B	4.95±0.58B	1(1.04)	0.00	X12
No. 09131- No. 02210	7.01±0.20A	7.32±0.52A	1(1.09)	0.00	5.04±0.44B	5.05±0.69B	0.99	0.01	X12
No. 09131- No. 91032	6.96±0.25A	6.87±0.25A	0.94	0.06	4.58±0.52B	4.90±0.91B	0.90	0.10	X12
No. 09131- No. 03482	7.53±0.58A	7.84±0.18A	1(1.10)	0.00	4.89±0.29B	5.40±0.56B	0.82	0.18	X12
No. 02454- No. 19179	6.79±0.44A	6.46±0.49A	0.86	0.14	4.29±0.68B	4.42±0.84B	0.96	0.04	W13
No. 02454- No. 01201	6.55±0.40A	6.61±0.66A	1(1.04)	0.00	4.19±0.24B	4.92±0.32B	0.70	0.30	W13
No. 09131- No. 19179	7.42±0.15A	7.62±0.09A	1(1.09)	0.00	5.40±0.98B	5.12±0.50B	1(1.08)	0.00	W13
No. 09131- No. 01201	7.29±0.15A	7.07±0.50A	0.92	0.08	4.56±0.14B	5.11±0.24B	0.80	0.20	W13
No. 19004- No. 19179	7.66±0.17A	7.73±0.22A	1(1.02)	0.00	4.15±0.08B	4.25±0.15B	0.97	0.03	W13
No. 19004- No. 01201	7.36±0.52A	7.70±0.35A	1(1.09)	0.00	4.50±0.31B	5.12±0.42B	0.80	0.20	W13
No. 19004- No. 02210	6.76±0.28A	6.88±0.16A	1(1.03)	0.00	3.65±0.37B	3.83±037B	0.94	0.06	W13
No. 19004- No. 91032	7.03±0.27A	7.19±0.22A	1(1.05)	0.00	4.15±0.33B	4.35±0.17B	0.92	0.08	W13
No. 19004- No. 03482	6.85±0.38A	6.98±0.48A	1(1.04)	0.00	3.60±0.16B	3.88±0.10B	0.91	0.09	W13
Qing662- No. 19179	7.30±0.53A	7.30±0.44A	0.97	0.03	4.99±0.44B	5.08±0.43B	0.97	0.03	W13
Qing662- No. 01201	5.66±0.35a	5.47±0.60a	0.82	0.18	4.44±0.11b	4.43±0.58b	1(1.05)	0.00	W13
Qing662- No. 02210	5.84±0.61a	5.67±0.66a	0.93	0.07	4.25±0.44b	4.51±0.18b	0.82	0.18	W13
Qing662- No. 91032	8.09±0.30A	7.33±0.42A	0.82	0.18	4.36±1.44B	4.94±0.44B	0.81	0.19	W13
Qing662- No. 03482	8.53±0.08A	8.32±0.17A	0.95	0.05	4.84±0.44B	5.02±0.49B	0.95	0.05	W13

^a^: “L” denotes large-seed lines; “S” denotes small-seed lines.

^b^: Rows followed by the same capital letter indicate no significance at the 0.01 probability level based on a Duncan test; rows followed by the same lowercase letter indicate no significance at the 0.05 probability level based on a Duncan test.

^c^: The large estimated maternal effects (> 1) were primarily due to the existence of the ultra-high and ultra-low seed weight parental individuals.

### Maternal genotype and cytoplasm effects on seed weight

There was a significant difference in seed weight between the large-seed line × small-seed line cross and small-seed line × large-seed line cross of the F_1_ seeds (Table 2). However, in the F_2_ generation (F_1_ plant) derived from reciprocal crosses of F_1_ seeds, seed weight showed no significant difference for most of the combinations and was between the parental values (Table 3), suggesting that seed weight was mainly controlled by maternal genotype, whereas the cytoplasmic effect had only minor or no influence.

**Table 3:** The seed weights of reciprocal crosses of the F_2_ generation derived from the F_1_ seeds.

Parental lines (L-S)	F_2_(L×S)^a^	F_2_(S×L)	Experiment code
No. 02454- No. 02210^b^	3.97±0.49a	3.78±0.32a	W13
No. 02454- No. 91032	4.31±0.40a	4.38±028a	W13
No. 02454- No. 03482	4.72±0.62a	4.72±0.32a	W13
No. 09131- No. 02210	3.66±0.22a	3.61±0.16a	W13
No. 09131- No. 91032	3.92±0.20a	3.99±0.58a	W13
No. 09131- No. 03482	4.39±0.35a	4.57±0.36a	W13
No. 02454- No. 19179	4.74±0.51a	4.52±1.19a	W14
No. 02454- No. 01201	4.31±0.39a	4.24±0.19a	W14
No. 09131- No. 19179	4.50±0.50a	4.63±0.28a	W14
No. 09131- No. 01201	4.05±0.19a	4.00±0.19a	W14
No. 19004- No. 19179	4.54±0.37a	4.47±0.36a	W14
No. 19004- No. 01201	4.17±0.40a	4.12±0.28a	W14
No. 19004- No. 02210	3.95±0.31a	3.75±0.40a	W14
No. 19004- No. 91032	4.23±0.22a	4.08±0.27b	W14
No. 19004- No. 03482	4.31±0.37a	4.32±0.22a	W14
Qing662- No. 19179	4.15±0.27a	4.38±0.37b	W14
Qing662- No. 01201	4.08±0.18a	4.13±0.34a	W14
Qing662- No. 02210	3.93±0.44a	3.85±0.51a	W14
Qing662- No. 91032	3.87±0.23a	3.85±0.30a	W14
Qing662- No. 03482	4.07±0.16a	4.14±0.19a	W14

^a^: “L” denotes large-seed lines; “S” denotes small-seed lines.

^b^: Rows followed by the same lowercase letter indicate no significance at the 0.05 probability level based on a Duncan test.

To quantitatively estimate the contributions of the maternal genotype and cytoplasmic effects on seed weight, an embryo-cytoplasm-maternal (GoCGm) model for diploid seeds was employed. The components of main genetic variance (*V*_*G*_ = *V*_*A*_+ *V*_*D*_ + *V*_*C*_ + *V*_*Am*_ + *V*_*Dm*_), *GE* interaction variance (*V*_*G*_ = *V*_*AE*_+ *V*_*DE*_ + *V*_*CE*_ + *V*_*AmE*_ + *V*_*DmE*_), and residual *V*_*e*_ are summarized in Table 4. The results showed that seed weight was mainly controlled by the main genetic effects (74.8%), whereas the influence of *GE* interaction effects was relatively small (6.4%). Among the effects of three sets of genetic systems (the embryo, cytoplasm and maternal plant), the seed weight in rapeseed was mainly controlled by the maternal genotype (*V*_*Am*_ + *V*_*Dm*_ + *V*_*AmE*_ + *V*_*DmE*_), accounting for 68.80% of *V*_*G*_, whereas embryonic and cytoplasmic effects, accounting for 12.28% and 0.15% of *V*_*G*_, respectively, had little influence on seed weight. Overall, these results indicated that seed weight was mainly controlled by the maternal genotype and that the cytoplasmic effect was small.

**Table 4:** Estimation of genetic variance components for seed weight in rapeseed.

Parameter	Variance	Parameter	Variance
*V*_*A*_	0.070**	*V*_*AE*_	0.014
*V*_*D*_	0.015	*V*_*DE*_	0.024**
*V*_*C*_	0.001**	*V*_*CE*_	0.000
*V*_*Am*_	0.483**	*V*_*AmE*_	0.000
*V*_*Dm*_	0.179**	*V*_*DmE*_	0.026**
		*V*_*e*_	0.188**

*V*_*A*_, embryo additive variance; *V*_*D*_, embryo dominance variance; *V*_*C*_, cytoplasmic variance; *V*_*Am*_, maternal additive variance; *V*_*Dm*_, maternal dominance variance; *V*_*AE*_, embryo additive interaction variance; *V*_*DE*_, embryo dominance interaction variance; *V*_*CE*_, cytoplasmic interaction variance; *V*_*AmE*_, maternal additive interaction variance; *V*_*DmE*_, maternal dominance interaction variance; *V*_*e*_, residual variance

** Significantly different at the 0.01 level.

## Discussion

The heterozygosity of each SSR marker was very low, suggesting that all nine of inbred lines are highly pure lines. Only nine rapeseed lines had a gene diversity (0.41) and PIC (0.35) values similar to those of 109 rapeseed lines from five world major rapeseed growing countries, which exhibited gene diversity of 0.43 and PIC of 0.37 [30]. The comparably abundant genetic diversity in the nine rapeseed inbred lines was primarily due to the broad seed weight diversity and different genetic backgrounds. The low kinship coefficients (78% of kinship coefficients were 0) suggested that most of the lines were unrelated or only weakly so, providing additional evidence that the nine inbred lines have broad genetic diversity.

In the present study, seed weight showed an extremely significant correlation and coordinated variation with seed size (including seed diameter, seed surface area and seed volume), but it showed no significant correlation with bulk density, suggesting that the weight of a seed is determined by its size rather than its bulk density. This is the first report on the morphological basis for seed weight variation in rapeseed, and the results are basically in agreement with studies in other crops. In rice, grain volume was found to be the major contributor (77%) to final grain weight variation [31]. Grain weight is also determined by factors that affect grain volume in wheat [32].

In the present study, the large-seed lines had, on average, 46.63% and 47.76% more cells than the small-seed lines in the seed coat and cotyledon, respectively, whereas these cells were, on average, only 24.93% and 22.80% bigger, which suggested that cell number was more tightly correlated with final seed weight. Moreover, correlation analysis showed that the traits affecting seed weight could be ranked in the following order: seed coat cell number > cotyledon cell number > seed coat cell size > cotyledon cell size. This is the first report on the cytological influence on seed weight variation in rapeseed. Similar phenomena have been observed in other crops. Lemontey et al. (2000) found that cotyledon cell number was the major factor affecting mature size because cell number appeared to be more tightly correlated with mature seed dry weight (r^2^ = 0.833, p < 0.001 for cotyledon cell number and seed dry weight; r^2^ = 0.345, p = 0.003 for cotyledon cell volume and seed dry weight) in five pea varieties with large differences in mature seed weight. The cell numbers in the seed coat and hypocotyl are the major factors that contribute to the seed length variations [33] of the two *Arabidopsis* strains, causing differences in seed sizes (i.e., the large-seed line had an average of 10% and 18% more cells than the small-seed line in the seed coat and hypocotyl, respectively, whereas these cells were, on average, only 6% and 4% longer, respectively).

Nine highly pure inbred lines with broad seed weight variation and different genetic backgrounds were used to study maternal and xenia effects. Improved materials and methods with minimal environmental influence led to the first accurate estimation of the maternal and xenia effects on rapeseed seed weight. Notably, the maternal effect value was estimated to be 0.93, indicating that the weight of the F_1_ hybrid seeds was mainly controlled by the maternal parent (93%). In the F_2_ generation derived from reciprocal crosses of F_1_ seeds, the difference in seed weight disappeared, which suggests that the weight of seeds was mainly controlled by maternal genotype, whereas the cytoplasmic effect had little or no influence. Moreover, the components of main genetic variance analysis [8, 29] confirmed that seed weight was mainly controlled by the maternal genotype, and the xenia and cytoplasmic effects were found to be small. In conclusion, we propose that the weights of seeds are mainly controlled by the maternal genotype, that is, the phenotype of the offspring is not determined by the genotype of the seed itself but, rather, is mainly controlled by the maternal genotype. This important discovery lays a foundation for genetic and breeding studies of seed weight in rapeseed.

We concluded that cell number was the major contributor to seed weight variation, and previous studies [33, 34] have shown that maternal effects mainly control cell number variation, which might implies that seed weight is mainly controlled by maternal effects. Our studies provide direct experimental evidence for the general rule that the weights of seeds are mainly controlled by maternal genotype in rapeseed. However, only a few cloned genes, such as *CYP78A5* [35], *ARF2* [36], *TTG2* [37], *AP2* [38–40], and *EOD3/CYP78A6* [41], acted maternally to regulate seed size/weight in *Arabidopsis*, whereas most of the cloned genes acted non-maternally to control seed size/weight [5]. This difference might occur because most of these cloned seed weight genes were identified by the analyses of *Arabidopsis* mutants, whose mutant loci are largely different with the natural variation [42]. In addition, most of these cloned seed weight/size genes involved the seed itself, i.e., maternal integument, embryo and endosperm development and growth. Therefore, seed weight research in rapeseed should focus on the maternal tissue rather than the embryo/endosperm, which will lead to a new area of exploration in seed weight research.

## Supporting Information

S1 Table The 487 rapeseed inbred lines/cultivars. (XLS)

S2 Table Details of the 101 SSR markers. (XLS)

S3 Table Nei's genetic distance matrix obtained from the frequencies of 101 SSR markers. (XLS)

S4 Table Kinship matrix for the nine inbred lines. (XLS)
